# Further Evidence for in Utero Transmission of Equine Hepacivirus to Foals

**DOI:** 10.3390/v11121124

**Published:** 2019-12-05

**Authors:** Stephane Pronost, Christine Fortier, Christel Marcillaud-Pitel, Jackie Tapprest, Marc Foursin, Bertrand Saunier, Pierre-Hugues Pitel, Romain Paillot, Erika S. Hue

**Affiliations:** 1 LABÉO Frank Duncombe, 14280 Saint-Contest, France; christine.fortier@laboratoire-labeo.fr (C.F.); Pierre-Hugues.PITEL@laboratoire-labeo.fr (P.-H.P.); Romain.PAILLOT@laboratoire-labeo.fr (R.P.); erika.hue@laboratoire-labeo.fr (E.S.H.); 2 BIOTARGEN EA7450, UNICAEN, NORMANDIE UNIV, 14000 Caen, France; 3 RESPE, 14280 Saint-Contest, France; c.marcillaud-pitel@respe.net; 4 Laboratory for Animal Health in Normandy, French Agency for Food, Environmental and Occupational Health & Safety (ANSES), 14430 Goustranville, France; jackie.tapprest@anses.fr; 5 Clinique équine de la Boisrie, 61500 Chailloué, France; marc.foursin@orange.fr; 6 Structural Virology Unit—CNRS UMR 3569, Institut Pasteur, 75015 Paris, France; bertrand.saunier@pasteur.fr

**Keywords:** non-primate hepacivirus, equine hepacivirus, in utero transmission, horse, fetuses

## Abstract

(1) Background: Equine hepacivirus (EqHV), also referred to as non-primate hepacivirus (NPHV), infects horses—and dogs in some instances—and is closely related to hepatitis C virus (HCV) that has infected up to 3% of the world’s human population, causing an epidemic of liver cirrhosis and cancer. EqHV also chronically infects the liver of horses, but does not appear to cause serious liver damages. Previous studies have been looking to identify route(s) of EqHV transmission to and between horses. (2) Methods: In this retrospective study, we sought to evaluate the prevalence of vertical transmission taking place in utero with measuring by quantitative RT-PCR the amounts of EqHV genome in samples from 394 dead foals or fetuses, paired with the allantochorion whenever available. (3) Results: Detection of EqHV in three foals most likely resulted from a vertical transmission from the mares to the fetuses, consistent with the in utero transmission hypothesis. In support of this observation, the presence of EqHV genome was found for the first time in two of the allantochorions. (4) Conclusions: As seemingly benign viruses could turn deadly (e.g., Zika flavivirus) and EqHV happens to have infected a significant proportion of the world’s horse herds, EqHV infectious cycle should be further clarified.

## 1. Introduction

**Figure 1:**
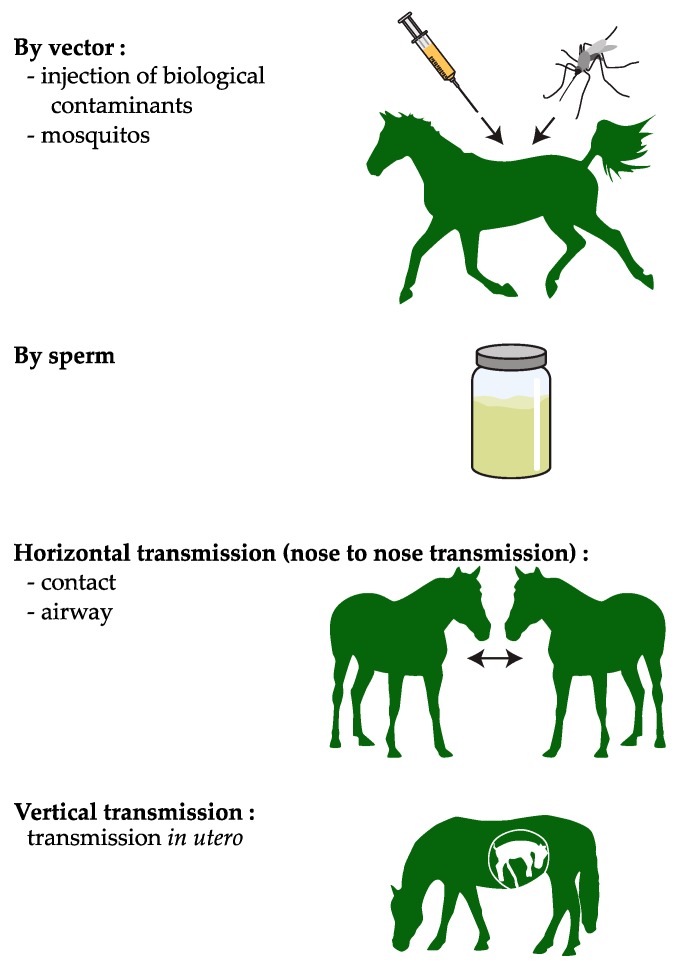
Hypotheses about the different modes of transmission by equine hepacivirus (EqHV) (according to [11]).

Since Burbelo et al. [1] first reported the infection of horses by an equine hepacivirus (EqHV) in 2012, the presence of this non-primate hepacivirus (NPHV) has been described in the equine populations of the five continents [1,2,3,4,5,6,7,8,9]. Depending on the geographic locations, the prevalence of EqHV measured by quantitative reverse transcriptase PCR (RT-PCR) varies from less than 1 to over 10 percent [2]. Among all hepaciviruses recently discovered in different animal species, EqHV displays the highest genomic homology to hepatitis C virus, which chronically infects humans in the liver. The study of hepaciviral infections in equids could shed some light on the physiopathology of HCV [10]; conversely, knowledge on HCV could also help investigating EqHV pathogenicity and elucidating its transmission route(s) (Figure 1).

A recent study performed in an area where EqHV is endemic in horses failed to detect the virus in a sample of over 5000 mosquitoes, making the latter an unlikely vector of this virus [12]. Other studies have reported the detections of EqHV-specific antibodies and/or of viral genome in serum, tissue samples and, lastly, in cerebrospinal fluid [13]. Detection and replication of EqHV genome in different organs of adult horses, such as liver, spleen, cerebellum, and lungs were also reported following the experimental infections [9,14,15]. Despite recent progress, EqHV tissue tropism remains largely uncharacterized in non-experimental conditions and little is known about the presence of the EqHV in equine fetuses. By comparison, most HCV infections in young children result from vertical transmission [16]. The contamination could take place during pregnancy or upon delivery [17,18], but its exact timing and mechanisms are not fully understood. Vertical transmission of EqHV in horses has started to be investigated only recently. In one study, the presence of EqHV viral RNA was measured in serum samples from 20 mare-foal pairs [19]. Evidence of transmission to the foal was reported for only one mare, and no potential route of transmission of the virus, such as intrauterine or postpartum transfer was identified [19].

The purpose of the present study is, by measuring the amount of viral genome in samples of a large population of foals deceased during the perinatal period (aborted fetuses, stillborn foals, death occurring during the first week of life), to evaluate the incidence of EqHV vertical transmission by the intrauterine route. Genomic sequence analyses showed that infections of paired mare and dead foal were caused by identical EqHV strains, demonstrating the existence of an in utero vertical transmission. 

## 2. Materials and Methods

The study and all animal work involved received ethical approval from the LABÉO Frank Duncombe ethical advisor (LFD-CE-07/2012, 2012). Samples were collected by equine veterinary practitioners according to a high standard of veterinary care. 

### 2.1. Necrospy and Histological Analysis

Specimens from foals deceased during the perinatal period were collected from French stud farms according to a standard protocol [20] and shipped at +4 °C to the Anses’ Necropsy Center (Laboratory for Animal Health in Normandy, France). As this is a retrospective study, information about the time lapse between delivery and autopsy of the fetuses may not be accurate; nevertheless, practitioner’s good practices include a reasonable routing time. Most samples were intended for routine bacteriological cultures, equine herpesvirus 1 (EHV-1), and equine viral arteritis (EVA) PCR analysis and histopathological analysis [21]. For owners who wished necropsy be performed on dead foals’ placentas, the latter were shipped using same guidelines: the samples were transported at +4 °C by a carrier and delivered within half an hour to LABÉO sample center; the samples were processed by the analytical services upon receipt.

In total, 394 tissue samples from aborted fetuses/neonatal foal deaths collected between January 2013 and December 2016 were used for this retrospective study. More information was available for 201 samples which allows to characterize the population: 159 fetuses with a median age of 8.75 months (7.25–9.50) and 42 foals with a median age of 24 h (4–72). In 2016, no sample was tested positive out of 130. During that year, 35 serum samples from mare who had aborted were also obtained and were all found negative. Serum was also obtained from foals of Cases #1 and #3 (cf. lower).

### 2.2. Molecular Detection and Characterisation of the EqHV Strains

#### 2.2.1. Nucleic Acid Extraction and Quantitative RT-PCR

RNA was extracted from 25 mg of homogenized organ tissue (liver + lung mixture, or allantochorion) with RNeasy Mini Kit (Qiagen, Courtaboeuf, France) or from 140 µL of serum with QIAmp viral RNA Mini kit (Qiagen, Courtaboeuf, France) according to the manufacturer’s instructions, and eluted in a final volume of 50 µl elution buffer (Qiagen, Courtaboeuf, France). After performing the originally planned tests, the extracted RNAs were stored at −80 °C in a temperature-monitored freezer until further use.

Quantitative RT-PCR was performed with One Step Prime Script RT-PCR kit (Takara, Ozyme, France) according to the manufacturer’s instructions and adapted from Burbelo et al. [1] on a StepOne™ Real-Time PCR system (Life Technologies, Saint-Aubin, France) [2]. Quantitative RT-PCR were performed with primers Qanti-5UF1, Qanti-5UR1, and probe 5’-FAM-CCACGAAGGAAGGCGGGGGC-BHQ1-3’ [1] and with a second pair of primers (Sau5UF 5’-TCGAGGGAGCTGRAATTCGT-3’, Sau5UR 5’-GCCCTCGCAAGCATCCTATC-3’), as previously described [2]. Thermal cycling proceeded at 42 °C for 5 min, 95 °C for 10 s, followed by 45 cycles: 95 °C for 5 s and 60 °C for 34 s. Fluorescence was measured at the end of each annealing/elongation step (60 °C). Data were analyzed using the StepOne™ software, version 2.2.2 (Life Technologies, Saint-Aubin, France). The limit of detection was 3.5 × 10^2^ genome copies/mL for serum and 7.7 × 10^3^ genome copies/g for tissue. 

#### 2.2.2. Sequencing and Phylogenic Analysis

Sequencing of 5’-untranslated region (5’UTR), non-structural protein 3 (NS3), and non-structural protein 5B (NS5B) amplimers was performed by Biofidal (Vaulx-en-Velin, France) with a Phusion Hot start II (Fisher Scientific, Illkirch, France) [2]. All sequences were deposited in GenBank (accession numbers MN229470-MN229484, KX239312-KX239466, KT175006-KT175040, as described in Supplementary Table S1). The concatenated sequences were obtained by joining: (i) 5′UTR, NS3, and NS5B regions (598 bp) for strains used in Figure 4A,B; and, (ii) 5′UTR and NS5B regions (472 bp) for strains used in Figure 4C.

Phylogenetic trees of nucleotide sequence alignments were created using the neighbor-joining method based on the Jukes-Cantor model of MEGA5 [22], as previously described [2]. Finally, all NS5B sequences used to build the tree were converted into a Nexus format using EMBOSS Seqret [23] and a median joining network was built using PopART (Population Analysis with Reticulate Trees) software, version 1.7 [24,25].

## 3. Results

### 3.1. Prevalence of EqHV in Perinatal Foal Deaths

Among 394 tissue samples collected from aborted fetuses, stillborn foals, or neonatal foal deaths samples, 3 (0.76%) were found positive for EqHV by quantitative RT-PCR (Table 1); 1/84 in 2013, 1/131 in 2014, 1/49 in 2015, and none out of 130 in 2016. The positive results were confirmed by nucleotide sequencing. The prevalence of EqHV viral RNA detected in this study contrasts with that of 6% previously observed in serum samples of French horses [2], suggesting that most transmissions occur via other paths, as reported in other studies [19].

In Case #1, EqHV genome was first detected in liver + lung samples from foal #1 (FR-Eq73 Liver-Lung) with a viral load of 2.3 × 10^7^ copies/g. Necropsy and histological analyses concluded to the lack of evidence for an overt viral infection; i.e., no significant lesion was observed in these two organs. This mare foaled again in 2015 (foal #2). No sample was available from the mare, but the presence of EqHV genome (FR-Eq84 Serum) was detected in serum from this foal.

In Case #2, EqHV was detected in both liver + lung necropsy sample and in the allantochorion (FR-Eq69 Liver-lung and FR-Eq69 Allanto in 2014) with a viral load of 2.4 × 10^7^ and 1.5 × 10^4^ copies/g, respectively. Necropsy and histological analyses concluded to the lack of lesion reminding a viral infection; no overt lesion was observed in these two organs. The mare (FR-Eq74) was not tested at the time of foaling but 10 months later and the serum sample was found positive (7.7 × 10^7^ copies/mL). 

In Case #3, 3.9 × 10^4^ copies/g of EqHV genome (FR-Eq70 in 2015) was detected in the allantochorion from a mare that foaled normally. Necropsy and histological analyses of the allantochorion concluded to a lack of histopathological signs of a viral infection; no obvious lesion was observed. Two months later, serum from the foal was tested negative while the maternal serum sample was still weakly positive with a viral load of 1.8 × 10^3^ copies/mL (FR-Eq85 in 2015).

Overall, the viral load measured in serum samples (1.8 to 7.7 × 10^7^ copies/mL) or liver + lung biopsies (2.3 to 2.4 × 10^7^ copies/g) were about 10^3^ higher than in allantochorion (1.5 to 3.9 × 10^4^ copies/g).

### 3.2. Vertical Transmission of EqHV from Mare to Foal

The 5′UTR, NS3, and NS5B regions were sequenced in all EqHV positive samples, with the exception of the two allantochorions, in which amplification of NS3 region failed. 

**Figure 2:**
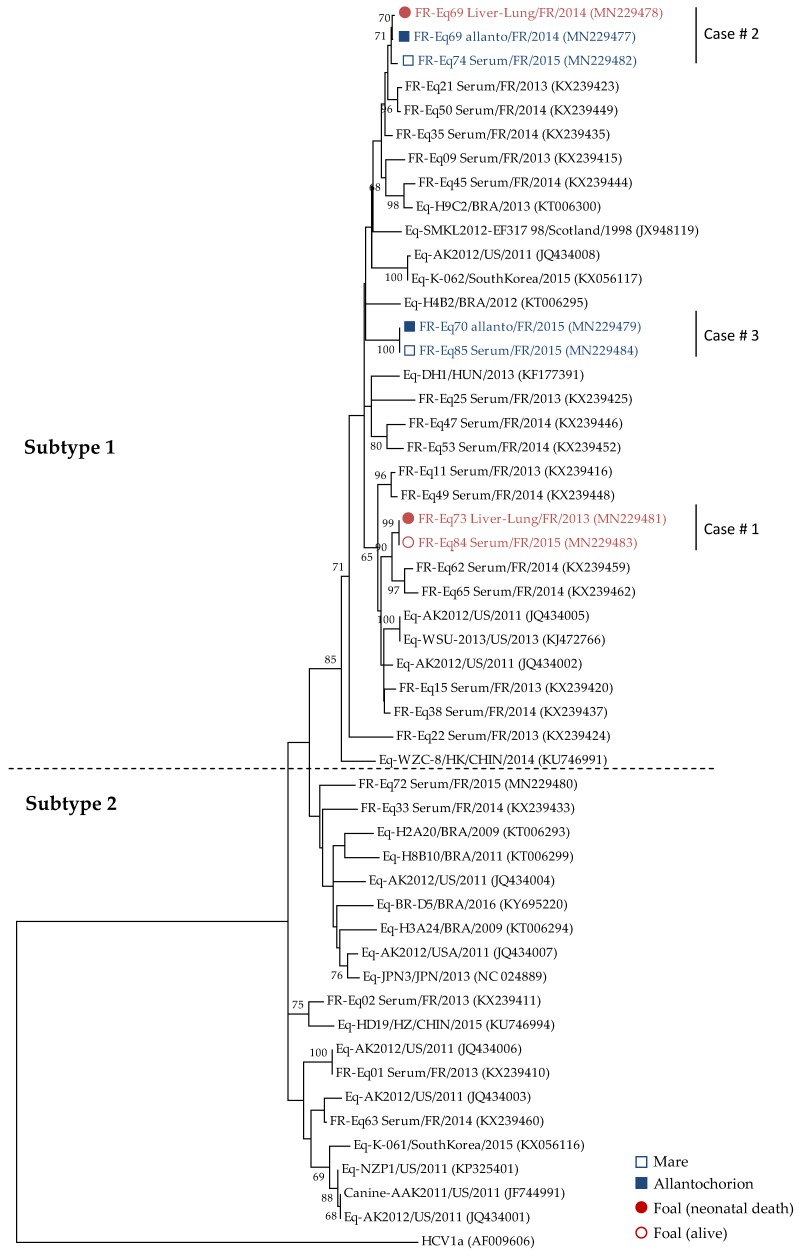
Phylogenetic analysis of equine hepacivirus NS5B sequences identified in horses. Neighbor-joining tree of partial nucleotide sequences from NS5B (259 bp) and corresponding region of a hepatitis C virus (HCV) genome (genotypes 1a). The tree was constructed with Jukes–Cantor model. A bootstrap was performed with a replicate rate of 500 (values ≥ 65% shown on branches).

Sequences from amplified NS5B region were obtained for the 7 samples reported in Table 1 and were incorporated in a phylogenetic tree including 46 other strains: (i). 20 that were selected to obtain a representative selection of EqHV strains from France in space and time according to Pronost et al. (2016, [2]); or, (ii). 26 samples from other countries (Supplementary Table S1) representing EqHV genetic diversity in the world so far. Albeit indirectly in Case #1, the NS5B phylogenetic tree strongly suggests a link between EqHV strains in samples from three mares and their respective foals or allantochorion (Figure 2). All EqHV strains from Cases #1 to #3 belong to subtype 1 [2] and these data were confirmed with the median joining network analysis (Supplementary Figure S1).

**Figure 3:**
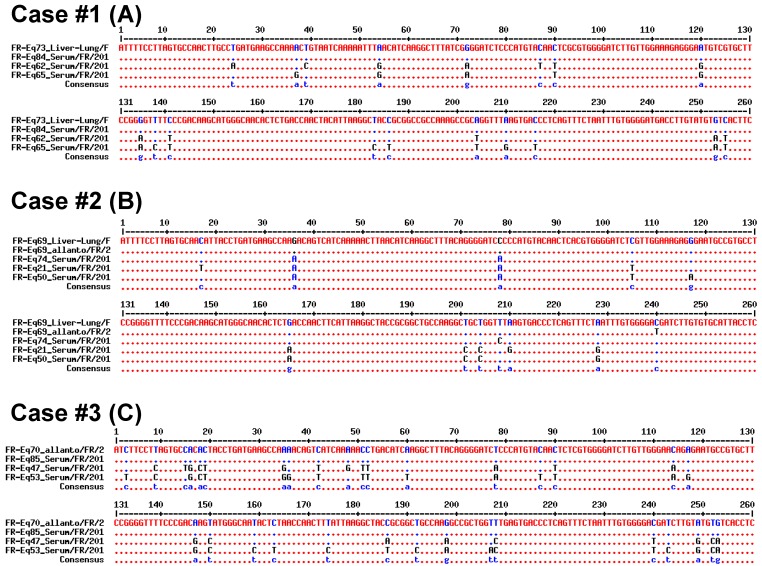
Alignment of NS5B sequences for samples Case #1 (**A**), Case #2 (**B**) and Case #3 (**C**) with the closest sequences identified by phylogenetic analysis (cf. Figure 2).

**Figure 4:**
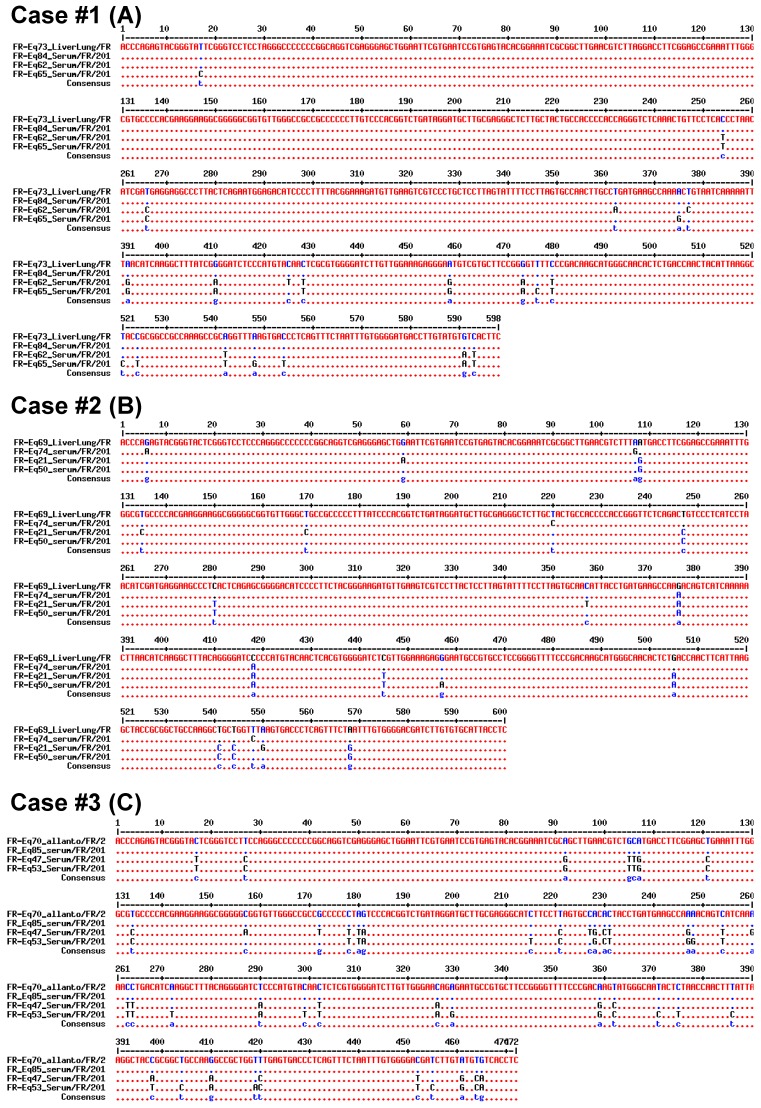
Alignment of concatenated sequences for samples Case #1 (**A**), Case #2 (**B**) and Case #3 (**C**) with those of the closest sequences identified by phylogenetic analysis (sequence numbers in Supplementary Table S1). Concatenated sequences were obtained from 5′UTR, NS5B, and NS3 sequences for A and B and from 5′UTR and NS5B for C.

For Case #1, no sample was directly available from the mare, but samples from her foals #1 (liver + lung) and #2 (serum) were analyzed; the two EqHV strains identified (FR-Eq73 and FR-Eq84) clustered with two other strains (FR-Eq62 and FR-Eq65). Alignment of the 260-bp NS5B sequences (Figure 3A) and the concatenated sequences (5′UTR + NS3 + NS5B; Figure 4A) show a total homology between the two strains. Sequence homologies with the two strains (FR-Eq62 and FR-Eq65) clustering in the phylogenetic tree (Figure 2) vary from 94.2% (15 nt difference) to 95.4% (12 nt) in the NS5B region and from 97% (18 nt) to 97.7% (14 nt) in the concatenated region. As a comparison, NS5B homology between French strains (with FR-Eq69 Liver-lung as reference strain) and strains isolated in other countries varied from 79% to 100%.

For Case #2, three different samples were analyzed: liver and lung biopsies from the foal (FR-Eq69 Liver-lung), the allantochorion (FR-Eq69 Allanto) and the maternal serum (FR-Eq74 Serum) drawn 10 months after foaling. Phylogenetic analysis evidenced only one base difference (99.6% homology) between the foal (liver + lung) and the allantochorion in the NS5B region (Figure 3B, nucleotide in position 240). No concatenated sequence was available for these two strains because NS3 sequencing failed for the allantochorion sample. These data suggest that these strains are closely related, since the number of discrepancies between analyzed regions is consistent with that of the mutations usually observed between isolates. Sequence homology with the strain obtained from the mare, ten months after abortion, varies from 98.8% (3 nt) in the NS5B region to 99% (6 nt) in the concatenated region when compared with the strain detected in the foal sample (FR-Eq69/liver-lung/FR/2014) (Figure 4B). Compared to this strain, the closest strains in the NS5B tree (FR-Eq21 and FR-Eq50; Figure 2) display a homology of respectively 96.5% (9 nt) and 96.9% (8 nt) in the NS5B region and 98.2% (11 nt) and 98.2% (11 nt) in the concatenated sequence (Figure 4B).

For Case #3, three samples were obtained: serum and allantochorion from the mare and serum from her foal. The EqHV sequences in the allantochorion (FR-Eq70) and the serum (FR-Eq85) samples were analyzed (the foal serum was negative). No differences were observed in the NS5B sequences (Figure 3C) nor in the concatenated 5’UTR+NS5B sequences (Figure 4C). Compared to the sequences of the two closest strains (FR-Eq47 and FR-Eq53), sequence homologies varied respectively from 91.5% (22 nt) to 88.1% (31 nt) in the NS5B region and from 92.6% (35 nt) to 91.1% (42 nt) in the concatenated region.

## 4. Discussion

From a phylogenetic point of view, EqHV (or NPHV) is very closely related to HCV. Therefore, transmission paths reported for HCV in humans—or in chimpanzees prior to the international ban on experiments involving great apes—have also been partially investigated in horses (Figure 1). The parenteral transmission of NPHV was experimentally demonstrated by Ramsay et al. and by Scheel et al. [14,15]. The works of Postel et al. and, more recently, of Lu et al. described the presence of equine hepacivirus in biological products from horses [26,27,28]. These observations highlight the risk of contamination when these products are injected as is in horses, as observed until the end of the 1980s for HCV with human blood transfusion, leading to an epidemic that has infected up to 3% of the world’s human population [29]. EqHV is also believed to be transmitted directly by blood, like reported for two more-distantly-related equine pegiviruses, TDAV (Theiler’s disease-associated virus), and EPgV (equine pegivirus) [30]. Other hypotheses, previously described for other viruses, cannot be totally excluded, such as infections transmitted by mosquitos or, less unlikely, medical treatment with contaminated blood products or instruments [1,9,19,26,31]. Yet, plasma and antitoxin inoculations are unlikely to account for the high seroprevalence of NPHV and EPgV in horses [32], suggesting that other modes of transmission may exist.

In addition to the parenteral route, a mother-to-child (vertical) transmission has been observed in 5% of human hepatitis C cases [16,18]. In spite a high prevalence of EqHV infections worldwide [1,2,3,4,5,6,7,8,9], such occurrence appears infrequent in horses. One case of vertical transmission from a mare to her colt was reported in 2015 [19]. This study was carried out on 20 gestating mares, 4 of whom were infected with EqHV at the time of delivery. The presence of EqHV genome was also detected in umbilical cord blood and in the serum of one of the foals. To the authors’ knowledge, this was the first report suggesting the possibility of in utero infection by EqHV. The rate of transversal infections was surprisingly high in the stud farm where this study took place; hence, we sought to further evaluate the prevalence of EqHV vertical transmissions in samples collected between 2013 and 2016 from several stud farms in France.

Among almost 400 cases of foal perinatal death, for which samples had been harvested in our laboratory, only three new cases of possible vertical transmission were identified, confirming an anticipated low incidence of new EqHV infections by this route. In Case #2, the presence of EqHV genome in three different biological compartments: organs of the foal (liver + lungs), the allantochorion, and the serum from the mare, could support this interpretation. The same 5′UTR sequences were obtained from organs of the foal and allantochorion, while only one-base difference was identified between NS5B sequences (FR-Eq69). The viral load in the allantochorion was very low and no NS3 sequence was obtained, but in our hands NS5B sequence is the most discriminating between strains [2]. Unfortunately, no blood sample was drawn from the mare at the time of delivery, but serum had been obtained 10 months later (FR-Eq74).

In HCV-infected individuals, quasispecies are defined as a group of similar-yet-not-identical viral genetic variants evolving at a rate between 0.8 and 2 milli-substitutions per nucleotide per site per year and overall presenting less than 5% nucleotide difference between genomes [20,27,28]. However, Gather et al. found only one-nucleotide change between EqHV genomes in maternal serum, umbilical cord blood and serum from her foal [19]. This could result from a relatively short delay between EqHV transmission and foal delivery. Alternatively, EqHV quasispecies in horses could drift at a slower pace than HCV quasispecies in humans. Therefore, in Case #2, it is far from clear whether the delay in the mare’s sampling would entirely explain a 5-nucleotide difference with her foal’s samples.

However, the most compelling argument for in utero transmission comes from the high viral loads detected in tissue samples from the neonate foal. First, it is highly improbable that a transmission upon delivery would produce so many genome copies in only two days [14,15]. Second, a simple contamination of the foal samples by maternal blood is also very unlikely, both during gestation and upon delivery. Thus, in the latter case, the virion concentration of maternal origin should be much more diluted than observed. In the former case, allantochorion anatomy is such that antibodies barely cross the chorionic barrier, if at all [33]; let alone viral particles of probably 50–70 nm in diameter. This comes in contrast to women, whose syncytiochorial placenta is bathed in maternal blood with potentially easy transfer of related HCV (and virus specific antibodies) from the maternal circulation to the syncytiotrophoblast. On the contrary, the equine epitheliochorial placenta has six layers of maternal and fetal tissues between the two blood circulations; hence, EqHV would be unlikely crossing this barrier, unless a receptor required for viral entry or acting as virion carrier was expressed on the allantochorion [34]. Were it nevertheless the case, given the worldwide prevalence of EqHV infections in mares, the number of neonate foals contaminated should be much higher than what is observed. Therefore, unless a hypothetical receptor isoform is involved, variants of the virus could promote EqHV in utero transmission. Finally, if the virus replicated within the allantochorion itself, as our results suggest, viral loads in the fetus would not any longer result from a contamination by maternal virions. Instead, the allantochorion would become a likely source of transmission to the fetus. The most likely explanation for Case #2 is, therefore, an in utero transmission of EqHV to the fetus.

In utero transmission to the fetus is also supported by the results obtained in Case #1. As no maternal sample was available, a direct link could not be established between EqHV identified in the three animals. Yet, the sequences of two EqHV-positive samples, one from a dead foal (liver + lung), born in 2013, and one from an apparently-healthy foal (serum), born later in 2015, were genetically clustered (FR-Eq73 and FR-Eq84). A 100% sequence homology was even found between the two isolates, which strongly suggested a similar source of transmission over a two-year period, here from the mare to her two offspring. In Case #3, the presence of a low EqHV viral load in allantochorion was detected, which displayed a 100% sequence homology with that identified in the mare’s serum (5’UTR and NS5B region). A serum sample from her foal could be analyzed only two months after foaling, with a negative result. As the virus load in allantochorion was low, a hypothesis is that the foal cleared its infection within two months. The mare also presenting with a weak viral load upon delivering was perhaps clearing her own infection; if so, her colostrum probably contributed to the foal’s quick recovery. In the absence of serological data, this cannot be confirmed, but a link between virus load and the risk of fetal infection has recently been discussed for HCV [35]. 

Our data are in agreement with Gather et al. [19], who suggested for the first time the possibility of in utero transmission of EqHV. Yet, in their study, all four placentas recovered from the EqHV-positive mares were negative by quantitative RT-PCR. The lack of EqHV RNA detection in placenta could result from a very low viremia, a low test sensitivity or the region of sampling given the anatomical heterogeneity of horse allantochorion [33]. In previous studies, we have developed a new EqHV quantitative RT-PCR method with specific primers designed on the basis of the first available equine NPHV genome [2]. Similarly, most studies, including that of Gather et al. [19], use primers from Burbelo et al. [1], initially designed to detect NPHV from different species. At parturition, placentitis was observed in one case, for which no EqHV genome was detected in the foal, suggesting to the authors that vertical transmission of NPHV occurred without an infection and inflammation of the placental tissue itself. In our study, the absence of histological inflammation in two placentas of EqHV-positive foals is in agreement with this interpretation, with the difference that it also establishes for the first time the presence of the virus genome in placenta. These findings suggest that, in the fetus like in adult horses, the presence of the virus is not associated to overt macroscopic or histological lesions, independently of the viral load. Nevertheless it takes sometimes several years before the consequences of what is at first considered a benign infection are identified. This outlines the importance of identifying as many transmission routes as possible.

Routes of transmission identified for other members of the *Flaviviridæ* family infecting horses and humans account for most observed infections. Thus, West Nile virus (WNV) is transmitted by mosquitoes; even if in rare occurrences, additional routes of transmission have been reported (for a review, see [36]). For example, the first case of in utero WNV transmission was reported in a woman in 2002 [37]. Abortion cases because of Japanese Encephalitis virus and severe Dengue infections were also reported after in utero transmission [38,39]. Mother-to-child transmission of WNV via breast milk has also been described (for a review, see [36]). Lastly, an epidemic of fetal microcephaly developing during Zika virus infections of pregnant women has raised serious concerns in several parts of the world [40]. Occupational exposure were also reported by different studies, which led to important safety implications for persons who work in these area.

Recently, our team completed a study on nasopharyngeal swabs and detected the presence of EqHV genomes in 4 of the 93 samples analyzed [41]. It did not enable establishing whether the transmission involved the respiratory tract, yet pointed at a possible role of the oropharyngeal sphere, as recently suggested by Altan et al. with the detection of EqHV genomes in a pool of four swabs [29]. Other works aim to elucidate the chain of transmission, such as a vector (e.g., mosquitoes) or, even if not yet reported, sperm during insemination or natural breeding. Additional studies are necessary to confirm or refute an involvement of these pathways during the transmission of the virus.

## Figures and Tables

**Table 1:** Features of the three cases, for which EqHV genome was detected in foal and/or allantochorion samples, in France between 2013 and 2016. (n.a. = not applicable).

Cases (Year)	Subjects	Sampling	Life Status (/Birth)	Viral Loads	EqHV Strains
Case #1 (2013 & 2015)	Foal #1 (4 days)	Liver+Lung	Neonatal death (4 days)	2.4 × 10^7^ copies/g	FR-Eq73_Liver-Lung/FR/2013
	Foal #2 (2 months)	Serum	Alive (2 months)	1.1 × 10^7^ copies/mL	FR-Eq84_Serum/FR/2015
Case #2 (2014)	Foal (2 days)	Liver+Lung	Neonatal death (2 days)	2.3 × 10^7^ copies/g	FR-Eq69_Liver-Lung/FR/2014
	Mare (5 years)	Allantochorion	n.a.	1.5 × 10^4^ copies/g	FR-Eq69_Allanto/FR/2014
		Serum	Alive (10 months)	7.7 × 10^7^ copies/ml	FR-Eq74_Serum/FR/2015
Case #3 (2015)	Foal (2 months)	Serum	Alive (50 days)	Negative	n.a.
	Mare (7 years)	Allantochorion	n.a.	3.9 × 10^4^ copies/g	FR-Eq70_Allanto/FR/2015
		Serum	Alive	1.8 × 10^3^ copies/mL	FR-Eq85_Serum/FR/2015

## Supplementary Materials

The following are available online at https://www.mdpi.com/1999-4915/11/12/1124/s1. Table S1: Characteristics of the different strains used for the phylogenetic tree of NS5B sequences. Figure S1: Median joining network based on the same nucleotide sequences as for the NS5B phylogenetic tree (Figure 2).
