# Supplementary material for: Further Evidence for in Utero Transmission of Equine Hepacivirus to Foals
Source: Viruses. 2019 Dec 5;11(12):1124. doi: 10.3390/v11121124 (PMC6950541; doi:10.3390/v11121124)
Supplement: Supplementary file 1 [file viruses-11-01124-s001.zip › Supplementary files/Supp table 1.pdf]

strain	sample type	Year	Country	GenBank accession number			Reference
				5'UTR	NS3	NS5B	
FR-Eq73_Liver-Lung/FR/2013	liver+lung	2013	France	KT175038	MN229474	MN229481	Pronost et al, 2016
FR-Eq69_Liver-Lung/FR/2014	liver+lung	2014	France	KT175035	MN229472	MN229478	Pronost et al, 2016
FR-Eq69_allanto/FR/2014	allantochorion	2014	France	KT175034	no amp	MN229477	Pronost et al, 2016
FR-Eq70_allanto/FR/2015	allantochorion	2015	France	KT175036	no amp	MN229479	Pronost et al, 2016
FR-Eq01_Serum/FR/2013	serum	2013	France	KT175006	KX239350	KX239410	Pronost et al, 2016
FR-Eq02_Serum/FR/2013	serum	2013	France	KT175007	KX239351	KX239411	Pronost et al, 2016
FR-Eq09_Serum/FR/2013	serum	2013	France	KT175011	KX239357	KX239415	Pronost et al, 2016
FR-Eq11_Serum/FR/2013	serum	2013	France	KT175012	KX239358	KX239416	Pronost et al, 2016
FR-Eq21_Serum/FR/2013	serum	2013	France	KT175015	KX239365	KX239423	Pronost et al, 2016
FR-Eq22_Serum/FR/2013	serum	2013	France	KT175016	KX239366	KX239424	Pronost et al, 2016
FR-Eq25_Serum/FR/2013	serum	2013	France	KT175017	KX239369	KX239425	Pronost et al, 2016
FR-Eq33_Serum/FR/2014	serum	2014	France	KT175018	KX239376	KX239433	Pronost et al, 2016
FR-Eq35_Serum/FR/2014	serum	2014	France	KT175019	KX239378	KX239435	Pronost et al, 2016
FR-Eq38_Serum/FR/2014	serum	2014	France	KT175022	KX239380	KX239437	Pronost et al, 2016
FR-Eq45_Serum/FR/2014	serum	2014	France	KT175023	KX239387	KX239444	Pronost et al, 2016
FR-Eq47_Serum/FR/2014	serum	2014	France	KT175024	KX239389	KX239446	Pronost et al, 2016
FR-Eq49_Serum/FR/2014	serum	2014	France	KT175025	KX239391	KX239448	Pronost et al, 2016
FR-Eq50_Serum/FR/2014	serum	2014	France	KT175026	KX239392	KX239449	Pronost et al, 2016
FR-Eq53_Serum/FR/2014	serum	2014	France	KT175027	KX239395	KX239452	Pronost et al, 2016
FR-Eq62_Serum/FR/2014	serum	2014	France	KT175028	KX239402	KX239459	Pronost et al, 2016
FR-Eq63_Serum/FR/2014	serum	2014	France	KT175029	KX239403	KX239460	Pronost et al, 2016
FR-Eq65_Serum/FR/2014	serum	2014	France	KT175030	KX239405	KX239462	Pronost et al, 2016
FR-Eq15_Serum/FR/2013	serum	2015	France	KT175014	KX239362	KX239420	Pronost et al, 2016
FR-Eq72_Serum/FR/2015	serum	2015	France	KT175037	MN229473	MN229480	Pronost et al, 2016
FR-Eq74_Serum/FR/2015	serum	2015	France	KT175039	MN229475	MN229482	Pronost et al, 2016
FR-Eq84_Serum/FR/2015	serum	2015	France	MN229470	MN229476	MN229483	this paper
FR-Eq85_Serum/FR/2015	serum	2015	France	MN229471	no amp	MN229484	this paper
NPHV_EF317_98/Scotland/1998	serum	1998	Scotland	JX948117	JX948118	JX948119	Lyons et al, 2012
DH1/HUN/2013	serum	2013	Hungary	KF177391, polyprotein gene			Reuter et 2014
AK2012_NPHV-NZP-1/US/2011	serum	2011	USA	JQ434001, polyprotein gene			Burbelo et al, 2012
AK2012_NPHV-G1-073/US/2011	serum	2011	USA	JQ434002, polyprotein gene			Burbelo et al, 2012
AK2012_NPHV-A6-006/US/2011	serum	2011	USA	JQ434003, polyprotein gene			Burbelo et al, 2012
AK2012_NPHV-B10-022/US/2011	serum	2011	USA	JQ434004, polyprotein gene			Burbelo et al, 2012
AK2012_NPHV-F8-06/US/2011	serum	2011	USA	JQ434005, polyprotein gene			Burbelo et al, 2012
AK2012_NPHV-G5-077/US/2011	serum	2011	USA	JQ434006, polyprotein gene			Burbelo et al, 2012
AK2012_NPHV-H10-094/US/2011	serum	2011	USA	JQ434007, polyprotein gene			Burbelo et al, 2012
AK2012_NPHV-H3-011/US/2011	serum	2011	USA	JQ434008, polyprotein gene			Burbelo et al, 2012
NZP1	serum	2011	USA	KP325401, complete genome			Scheel et al, 2015
Canine-AAK2011/US/2011	serum	2011	USA	JF744991, polyprotein gene			Kapoor et al, 2011
WSU-2013/US/2013	serum	2013	USA	KJ472766, polyprotein gene			Evanoff submitted
H4B2	serum	2013	Brazil	KT203940	/	KT006300	Figueiredo et al, 2015
H9C2	serum	2012	Brazil	KT203935	/	KT006295	Figueiredo et al, 2015
H2A20	serum	2009	Brazil	KT203933	/	KT006293	Figueiredo et al, 2015
H8B10	serum	2011	Brazil	KT203939	/	KT006299	Figueiredo et al, 2015
H3A24	serum	2009	Brazil	KT203934	/	KT006294	Figueiredo et al, 2015
1040/16-14565FG	serum	2016	Italia	KY695184	KY695202	KY695220	Elia et al, 2017
WZC-8/HK/China	serum	2014	China	KU747003	KU746997	KU746991	Lu et al, 2016
HD19/HZ/China	serum	2015	China	KU747006	KU747000	KU746994	Lu 2016
JPN3/JPN/2013_(NC_024889)	serum	2013	Japan	NC_024889, polyprotein gene			Baechlein et al, 2015
K-061	serum	2015	South Korea	KX056116, polyprotein gene			Kim, unsubmitted
K-062	serum	2015	South Korea	KX056117, polyprotein gene			Kim, unsubmitted
