# Supplementary figures and images for: Further Evidence for in Utero Transmission of Equine Hepacivirus to Foals
Source: Viruses. 2019 Dec 5;11(12):1124. doi: 10.3390/v11121124 (PMC6950541; doi:10.3390/v11121124)

Subtype 1

Case #3

Case #2

Case #1

Subtype 2

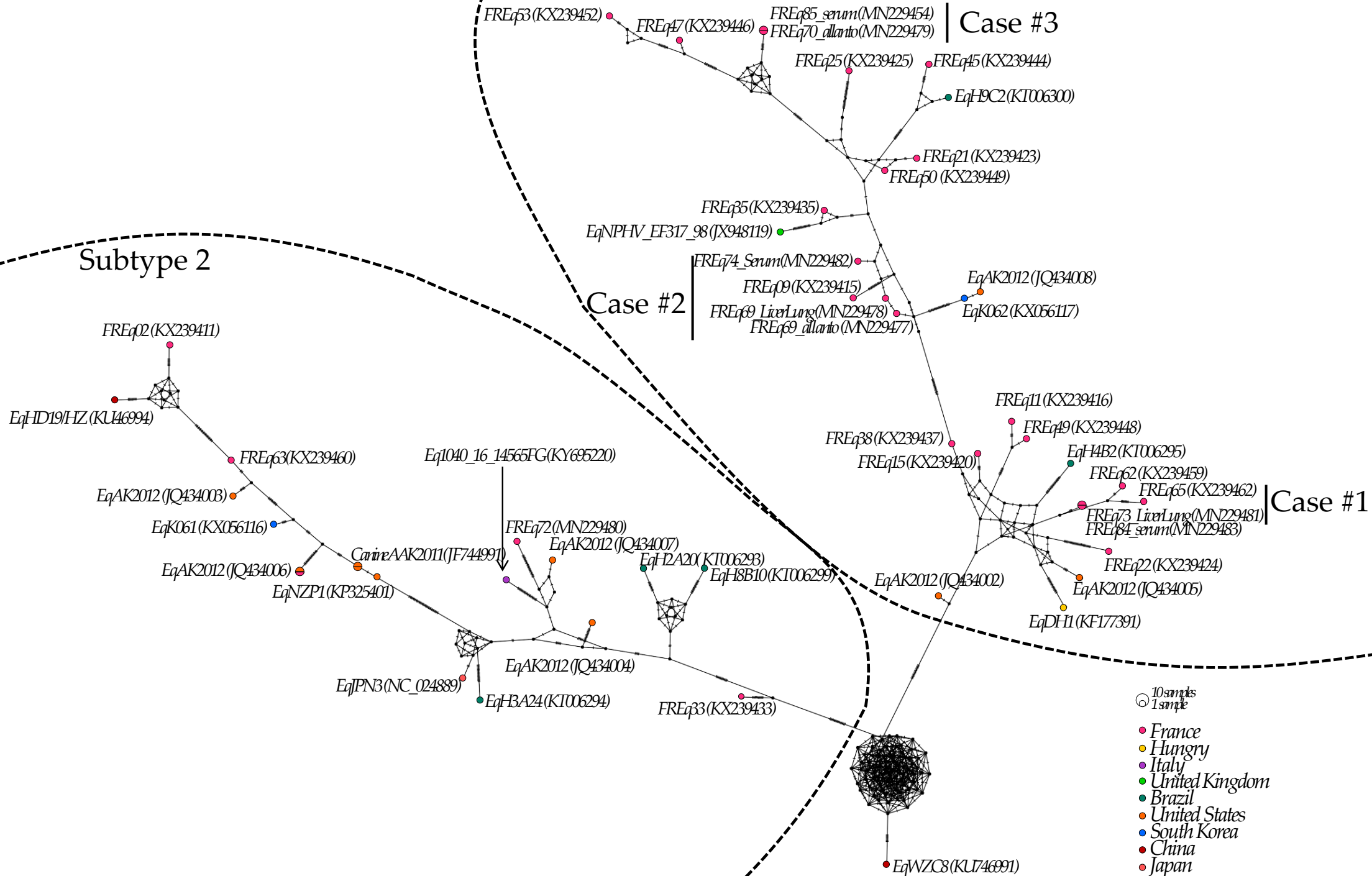

Supplement: Supplementary file 1 [file viruses-11-01124-s001.zip › Supplementary files/Supp_Figure 1.pdf]
